# Bacteremia in the red blood cells obtained from the cell saver in patients submitted to heart surgery

**DOI:** 10.1590/1518-8345.3092.3337

**Published:** 2020-08-31

**Authors:** Manuel Luque-Oliveros

**Affiliations:** 1 Universidad de Sevilla, Sevilla, Espanha.

**Keywords:** Blood Transfusion, Autologous, Bacteremia, Thoracic Surgery, Erythrocytes, Extracorporeal Circulation, Operating Room Nursing, Transfusão de Sangue Autóloga, Bacteriemia, Cirurgia Cardíaca, Eritrócitos, Circulação Extracorpórea, Enfermagem de Centro Cirúrgico, Transfusión de Sangre Autóloga, Bacteriemia, Cirugía Cardíaca, Eritrocitos, Circulación Extracorpórea, Enfermería de Quirófano

## Abstract

**Objective:**

to determine the microbiological characteristics of the red blood cells obtained with the cell saver in heart surgery patients on an extra-body circuit.

**Method:**

a cross-sectional and descriptive study conducted with 358 patients scheduled for heart surgery where the saver was used. Sociodemographic variables were collected, as well as from the saver and of the microbial identification in the re-infusion bag proceeding from the cell saver. Informed consent performed.

**Results:**

of the 170 GRAM+ bacteria isolations, the most frequent species were *Staphylococcus epidermidis* in 69% (n=138) of the cases and *Streptococcus sanguinis* with a report of 10% (n=20). Significant differences were found in the *Staphylococcus epidermidis* strain in patients with a Body Mass Index ≥25 (p=0.002) submitted to valve surgery (p=0.001). Vancomycin was the antimicrobial which resisted the *Staphylococcus epidermidis* strain with a minimum inhibitory concentration of >16 µg/ml.

**Conclusion:**

the microbiological characteristics of the red blood cells obtained after processing autologic blood recovered with the cell saver during heart surgery are of GRAM+ bacterial origin, the most isolated species being *Staphylococcus epidermidis*. Consequently, in order to reduce the presence of these GRAM+ cocci, an antibiotic should be added to the cell saver reservoir, according to a previously established protocol.

## Abstract (pt)

**Objetivo:**

determinar as características microbiológicas dos glóbulos vermelhos obtidos com o “cell saver” em pacientes submetidos à cirurgia cardíaca em circuito extracorpóreo.

**Método:**

estudo descritivo transversal com 358 pacientes de cirurgia cardíaca em que o “cell saver” foi utilizado. Foram coletadas variáveis sociodemográficas e identificação microbiana da bolsa de reinfusão do “cell saver”. Foi confirmado o Termo de Consentimento.

**Resultados:**

das 170 baterias isoladas GRAM+, as mais frequentes foram *Staphylococcus epidermidis* em 69% (n=138) dos casos e *Streptococcus sanguinis* com um registro de 10% (n=20). Diferenças significativas foram encontradas na cepa *Staphylococcus epidermidis* em pacientes com índice de massa corporal ≥25 (p=0,002) submetidos a cirurgia valvular (p=0,001). A vancomicina foi o antimicrobiano resistente à cepa *Staphylococcus epidermidis* com uma concentração inibitória mínima >16 µg/ml.

**Conclusão:**

as características microbiológicas dos glóbulos vermelhos obtidas após o processamento do sangue autólogo recuperado com o “cell saver” em cirurgia cardíaca são de origem bacteriana GRAM+, sendo a espécie mais isolada o *Staphylococcus epidermidis*. Portanto, para reduzir a presença desses cocos GRAM+, um antibiótico deve ser adicionado ao “cell saver”, de acordo com um protocolo previamente estabelecido.

## Abstract (es)

**Objetivo:**

determinar las características microbiológicas de los glóbulos rojos obtenidos con el “cell saver” en el paciente de cirugía cardíaca bajo un circuito extracorpóreo.

**Método:**

estudio descriptivo transversal con 358 pacientes programados para cirugía cardíaca donde se utilizó el “cell saver”. Se recogieron variables sociodemográficas e identificación microbiana de la bolsa de reinfusión procedente del “cell saver”. Consentimiento informado realizado.

**Resultados:**

de 170 aislamientos de bacterias GRAM+, las especies más frecuentes fueron el *Staphylococcus epidermidis* en el 69% (n=138) de los casos y el *Streptococcus sanguinis* con un reporte del 10% (n=20). Se encontraron diferencias significativas en la cepa *Staphylococcus epidermidis* en pacientes con índice de masa corporal ≥25 (p=0,002) sometidos a cirugía valvular (p=0,001). La vancomicina fue el antimicrobiano resistente a la cepa *Staphylococcus epidermidis* con una concentración mínima inhibitoria >16 ug/ml.

**Conclusión:**

las características microbiológicas de los glóbulos rojos obtenidos tras el procesamiento de sangre autóloga recuperada con el “cell saver” en cirugía cardíaca son de origen bacteriano GRAM+ siendo la especie más aislada el *Staphylococcus epidermidis*. Por ello, con el fin de reducir la presencia de estos cocos GRAM+ se debería añadir un antibiótico en el reservorio del “cell saver”, según un protocolo previamente establecido.

## Introduction

Invasive infections during heart surgery have been reported internationally^(^ 1 ^)^; in fact, bacterial contamination outbreaks have been described recently in the cooling-heating devices used to regulate the patient’s blood temperature during the extra-body flow through the closed water circuits^(^ 2 ^)^. New studies indicate the presence of other bacterial colonizations in the membrane oxygenators of the extra-body circuit itself, which compromises bacterial re-circulation in the patients during the time the extra-body machine is used^(^ 3 ^-^ 8 ^)^. Additionally, knowing that heart surgery is the medical specialty which consumes the most blood products^(^ 9 ^)^, the cell saver (CS) is used, a device which recovers blood losses from the surgical field and from the residual volume of the extra-body circuit to submit it to a “cleaning” process, obtaining a certain volume of red blood cells which is reinfused into the patient (re-infusion bag). In this device, bacterial growth was detected in 85% of the samples of recovered blood^(^ 10 ^)^ and in the samples of red blood cells to reinfuse GRAM+ bacterial commensals^(^ 11 ^)^, and even in other surgical procedures like traumatologic ones (scoliose in Pediatrics), where the CS was also used and the same commensals were detected in the re-infusion bag, but of the *staphylococcus* genus^(^ 12 ^)^.

The presence of bacteria in the saver, both in its recovery process and in its reinfusion phase, supposes a potential infection risk for the patient during the surgical procedure since these devices lack anti-bacterial protection^(^ 13 ^)^. This fact gains importance because the presence of bacteremia in the patient is associated with vascular accesses, prolonged hospitalizations, treatments in intensive care units, and administration of multiple broad-range antibiotics where immuno-suppresion is the most important comorbidity and where mortality is significant^(^ 14 ^)^.

Currently, considering the weight of the existing evidence and in the context of heart surgery, there is little evidence on the different types of bacteremia that can be contained in the red blood cell bag which is reinfused into the patient, proceeding from the cell saver, reason why we set ourselves the objective of determining the microbiological characteristics of the red blood cells obtained with the cell saver in the heart surgery patient on an extra-body circuit.

## Method

A cross-sectional and descriptive study conducted with patients scheduled for the heart surgery service in the period from May 1^st^ to October 1^st^, 2017, with its data obtained from the managerial request for the surgical demand, in Sevilla, Spain. Of all the patients submitted to heart surgery on an extra-body circuit with the use of the C.A.T.S^®^ *(Continuous Autologous Autotransfusion System, Fresenius)* cell saver, those over 18 years of age and who accepted to participate in the study after verbal introduction and explanation of the informed consent were included. Patients were excluded when the saver was not used according to the indications of the scheduled surgery. The exclusion criteria also included immuno-suppressed patients (infectious-contagious or carcinogenic diseases; for being contraindication measures to the use of the saver), and patients on anti-microbial therapy because it creates a bias in data collection. Before the surgery, the patients were conventionally and uniformly prepared: continuous hemodynamic monitoring, with general anesthesia in its three phases (induction, maintenance, and recovery) with the same drugs at a dose proportional to the patient’s weight and height.

The surgical approach was conducted homogeneously in all the patients: sternal dissection and opening by means of longitudinal sternotomy, cannulation of big blood vessels, similar perfusion protocols and extra-body flow techniques, and with similar surgical techniques employed during the surgery.

After finishing the surgery, the hemostasis of all the anastomosis was verified, and coagulation was reverted by means of protamine sulphate with a 1:1 ratio with heparin, where all the extubated patients were given the fast track protocol inherent to the post-anesthetic service.

The preparation and management of the saver in all the patients was undifferentiated: for circuit priming and purge 3,000 mL of crystalloids were used, combined with 30,000 IU of sodium heparin to avoid coagulation. The surgical field blood losses were recovered with a suction vacuum system which can be regulated to -20 cm H_2_O, as well as those from the residual volume in the extra-body circuit^(^ 15 ^)^, where it passed through a 25 µm filter into a reservoir and, after finishing the surgery, its processing or the wash cycle began to obtain the red blood cell bag which was reinfused into the patient. The perfusionist herself assembled and purged the saver.

The operating room conditioning (access and walking aisles, construction characteristics, medicinal gases, thermal isolation, safety conditions, equipment, and ventilation systems) complies with the necessary requirements for asepsis inherent to the type and risk level of the intervention. In addition, general inspections are performed in the operating room to detect environmental contamination according to the current standards.

The hospital’s Microbiology Service worked with the Becton Drive Bactec^TM^ 960 bacterial automated system to measure CO_2_ production from microbiologically active micro-organisms by means of spectrophotometric methods or fluorescence, where each vial is controlled continuously and detection is external; as the vials are not opened, the possibility of cross-contamination is eliminated. For microbial identification, bacterial phenotypic identification techniques were carried out under a seriated microbiological culture of red blood cells proceeding from the saver reinfusion bag. For the isolation and identification of the pathogen agent, 20 mL of red blood cells were previously extracted (after finishing the entire wash cycle and before reinfusion into the patient), where the first 10 mL were introduced in an anaerobics vial and the remaining 10 mL in an aerobics vial, performing the inversion technique. In the identification process of the bacterial strain, its morphology, growth, biochemical, and metabolic properties where observed and, when feasible, the involved micro-organism was identified. The porter himself sent the vials, and he employed asepsis techniques during the transfer, the send-receive time not exceeding 8 minutes.

The studied variables were; age, gender, scheduled surgical procedure, surgery time, body mass index, collected from the patient’s clinical record; recovered blood volume in the reservoir, erythrocyte concentrate volume, discarded remaining volume, and processing time, obtained through the information available in the CS; GRAM+ cocci, GRAM- cocci, GRAM+ bacillus, and GRAM- bacillus with isolation in their diversity, collected through microbiology. All collected data were recorded in our *ad hoc* document.

For data exploitation, the SPSS for Windows statistical program was used, expressing the categorical variables by means of percentages and of relative and absolute frequencies.

Finally, the continuous variables are presented through central tendency and standard deviation measures. Pearson’s chi-square test and Fisher’s exact test were used in the categorical variables, as it was found pertinent. Finally, the Student’s t test was applied to the continuous variables to determine whether there were statistically significant differences. A significant p value<0.05 and a 3% sampling error were set.

The studied complied with the principles set forth in the Declaration of Helsinki, in Organic Law of Personal Data Protection No. 15/1999 of December 13^th^, and in Royal Decree No. 1,720/2007, which develops it. All the patients were informed both verbally and through the informed consent form. This study obtained the formal approval of the hospital’s Commission on Ethics and Clinical Research.

## Results

Of the total 100% (n=358) of the patients scheduled for heart surgery, 56.1% (n=201) were selected for meeting the inclusion criteria, and 43.9% (n=157) were excluded because, in 41.7% (n=149) of the cases, the CS was not used, and because 2.2% (n=8) did not sign the informed consent form. The characteristics of the patients were the following: mean age of 65 years old, mainly men (74%) with a Body Mass Index of 24.2, the aortic valve surgery being the most common (65%) according to Table 1. The surgical times were the following: aortic valve, 242 minutes, tricuspid valve, 270 minutes, mitral valve, 260 minutes, and coronary bypass, 334 minutes.

**Table 1:** Characteristics of the patients (201) included in the study. Seville, Spain, 2017

Variable	Mean Fr (%)*	Standard deviation
**Age (years)**	65	8.9
30 to 45	7 (3.5)	
46 to 65	143 (71.1)	
>65	51 (25.4)	
**Gender**		
Male	149 (74.1)	
Female	52 (25.9)	
**Body Mass Index**	24	2.1
Low weight <18.5	1 (0.5)	
Normal weight 18.5 - 24.9	124 (61.2)	
Overweight 25 - 29.9	76 (37.3)	
Obese >30	2 (1)	
**Scheduled surgery**		
Aortic valve	131 (65)	
Tricuspid valve	2 (1)	
Mitral valve	28 (14)	
**Coronary bypass**	40 (20)	
1 vessel	19 (9)	
2 vessels	18 (9.5)	
3 vessels	3 (1.5)	

* Fr (%) = Frequency (percentages)

Regarding the cell saver values, the following figures were obtained: mean volume of 2,163 ml of recovered blood (Standard Deviation = 588.67), 602 ml of red blood concentrates (Standard Deviation = 177.49), 4,719 ml of discarded remnants (Standard Deviation = 1,282.01) in a mean processing time of 25 minutes (Standard deviation = 5.83).

With respect to the microbial identification, the coagulase-negative GRAM+ bacteria were the most frequently isolated in the *Staphylococcus epidermidis* species, with 69% (n=138) of the cases, followed by *Streptococcus sanguínis* (*S. viridans*) with a report of 10% (n=20) according to Table 2.

**Table 2:** Bacterial species identified in 85% of the red blood cell reinfusion bag proceeding from the cell saver. Seville, Spain, 2017

Bacterial group	Fr (%)*
**GRAM+ cocci**	
Staphylococcus	
*epidermidis*	138 (69)
*haemolyticus*	2 (1)
*hominis*	4 (2)
*caprae*	2 (1)
*simulans*	2 (1)
*cohnii*	2 (1)
Streptococcus	
*sanguinis* (*viridans*)	20 (10)
**GRAM- cocci**	
**GRAM+ bacillus**	
Corynebacterium	
*diphteroides*	7 (4)
**GRAM- bacillus**	
Bacteroides	
*fragilis*	2 (1)

* Fr (%) = Frequency (percentages)

In the relation of the *Staphylacoccus epidermidis* strain according to the profile of the studied patient, statistically significant differences were found with respect to the Body Mass Index (p=0.002) and to the surgical procedure performed (p=0.001), according to Table 3.

**Table 3:** Relation of the presence of *Staphylococcus epidermidis* with different characteristics of the studied patient (n=201). Seville, Spain, 2017

	Presence of *Staphylococcus epidermidis*		
Variable	Yes (n=138) Fr (%)	No (n=63) Fr (%)	p
**Age (years)**			0.16*
<65	101 (67.3)	49 (32.7)	
≥65	37 (72.5)	14 (27.5)	
**Gender**			0.31*
Male	99 (66.4)	50 (33.6)	
Female	39 (75)	13 (25)	
**Body Mass Index**			0.002^†^
<25	63 (50.4)	62 (49.6)	
≥25	75 (98.7)	1 (1.3)	
**Surgery**			0.001^†^
Valve	135 (83.8)	26 (16.2)	
Bypass	3 (7.5)	37 (92.5)	

* Pearson's Chi-Square;

† Fisher's Exact test. Significance level: p<0.05.

The antimicrobial which resisted the *Staphylococcus epidermidis* strain was Vancomycin, with a minimum inhibitory concentration of >16 µg/ml.

**Table 4:** Antimicrobial sensitivity of *Staphylococcus epidermidis*. Seville, Spain, 2017

Antimicrobials	Sensitivity (S*/I^†^/R^‡^)	Minimum Inhibitory Concentration (µg/ml)
Rifampycin Daptomycin Tigecycline Vancomycin Phosphomycin Linezolid Methicillin	S S S R I S R	<=0.015 <=0.25 <=0.5 >16 <=8 <=1 >2

* S = Sensitive;

† I = Intermediate;

‡ R = Resistant

## Discussion

The study data expose the presence of bacteria in the red blood concentrates of the reinfusion bag, with a high report of coagulase-negative GRAM+ bacteria in their *Staphylococcus epidermidis* species composing part of the cutaneous and mucous flora, where their identification sets forth the following dilemma among the professionals: contamination or real bacteremia?

Currently, in developed countries the vast majority of intra-hospital microbiological isolations in blood are due to GRAM+ cocci, including those due to coagulase-negative *Staphylococcus* ^(^ 16 ^)^ and, in some studies, it is the main cause of hospital bacteremia^(^ 17 ^)^. In a study, coagulase-negative *Staphylococcus* was identified in the concentrate obtained from the saver in 54.2% of the cases, reason why it was pointed out that the use of this device is not the best option in surgery. However, this study lacks the inclusion of an extra-body circuit and does not even expose the different types of isolated micro-organisms in the saver, since it only focused on isolating one of them^(^ 12 ^)^.

Likewise, in other studies GRAM+ bacteria were detected by blood extraction from the patient, concluding the hetero-resistance to certain antibiotics^(^ 14 ^,^ 18 ^)^, but its results include only hospitalized (not surgical) patients, reason why its prevalence is not demonstrated in the context of heart surgery for not using the saver.

It is to be recalled that bacterial infections are the leading cause of mortality due to blood transfusions^(^ 19 ^)^, as confirmed by the data from a research study^(^ 20 ^)^, which verified a significantly higher infection risk in the patients treated with a liberal transfusion policy than in those treated with a restrictive strategy (12.7% *vs* 10.6%). In this study it was concluded that a restrictive transfusion strategy for red blood cells was associated with a reduction in the infection risk (pneumonia, mediastinitis, infection of the surgical wound, and sepsis) if compared to the liberal transfusion strategy. However, they do not analyze its cause (type of originating bacteria) nor do they describe the type of red blood cell transfusion they perform, with the possibility of it being homologous or autologic, as the case of the saver.

In other studies^(^ 21 ^)^, in 83 samples the presence of *Staphylococcus epidermidis* was identified, thus concluding that this strain could be susceptible to causing infections in the bloodstream. However, the isolations were performed in patients with neoplastic pathologies, and not in the context of heart surgery.

On the other hand, the report of a research study^(^ 22 ^)^ states that *Staphylococcus epidermidis* is a well-known etiology of the endocarditis of prosthetic valves, and the one emerging from native valve endocarditis where they have been successfully treated with antibiotic therapy like Vancomycin. We disagree with this author because, in the study, Vancomycin showed resistance to the same strain >16 µg/ml.

Recent studies show that adding Vancomycin to the wash serum of the CS itself is effective on bacterial contamination, allowing for its elimination^(^ 2 ^)^ but, in these studies, the participants were scheduled for back spinal fusion surgery, did not use an extra-body circuit, and the study only determines the bacterial elimination in the saver reservoir without subsequently analyzing bacteremia in the red blood cell bag, as shown in our study.

We do agree with the studies which identified that *Staphylococcus epidermidis* was the most frequent micro-organism in heart surgery, where the unsaturation of different diagnostic and therapeutic measures taken by a multidisciplinary team had a favorable influence on the patients’ prognosis^(^ 23 ^)^. Other studies^(^ 24 ^)^ show the same results. However, they did not determine the isolation source or the antimicrobial sensitive to the strain.

Despite the improvements in the surgical techniques, in the materials, and in the design of the devices, the associated infection is still a relatively frequent and serious complication. Another research study^(^ 25 ^)^ shows that the infection is generally produced during the surgery starting from the patient’s cutaneous microbiota, when the micro-organisms colonize a device, grow on its surface forming a bio-layer which is determinant in the pathogenia of these infections, reason why we emphasize the importance of identifying the species in any isolation which is considered significant^(^ 26 ^)^ because GRAM+ cocci are increasingly involved in intra-hospital infections^(^ 27 ^)^.

Additionally, a recent multi-centric study conducted in 26 hospitals (PROBAC cohort) detected bacteremia episodes in the intra-cardiac devices due to GRAM+ cocci in 30.4% of the patients, a fact which undoubtedly evidences that the infection risk due to hematogenous seeding is present in these patients^(^ 28 ^)^, even with the possibility of triggering an infectious endocarditis (IE).

According to a new study, the origin of the IE episode could be attributed to diagnostic procedures in 10.52% of the cases, to intercurrent infectious processes in 42.11% of the cases, to other surgical procedures in 26.3% of the cases, and to unknown causes in 21.05% of the cases, with the need for surgical treatment in all the cases^(^ 29 ^)^. The fact that an IE is produced due to any of the aforementioned causes generates a considerable increase in the risk of the bacterial layer settling on the prosthetic materials, or of residual injuries and frequently on right cavities^(^ 30 ^)^, which becomes a serious form of the disease with high global mortality^(^ 29 ^)^. Nevertheless, these researchers did not determine the source of the bacteremia, nor did they establish the elements or components of the possible infection focuses, which is necessary to establish a clinical suspicion prior to providing an early diagnosis and treatment to the patients.

Up to the present day, conventional therapies with antimicrobial agents are used to treat and prevent the associated infections; however, the professionals disagree on the origin of the cardiac patient’s infections during the post-operative period, based on the existing data. Furthermore, recent research studies assert that, under certain conditions, the effectiveness and benefits for the patients from using the cell saver under extra-body circulation are ambiguous, with discrepancies among the studies or the patients^(^ 31 ^)^. Not to mention the current safety problems in cases of severe bacterial contamination where future studies will be needed to better determine how and when the cell saver is to be used, alongside with new blood conservation measures^(^ 32 ^)^.

In this context, our study adds additional information on a possible infection focus to the scientific knowledge, incorporating isolation frequencies of bacterial strains in the red blood cell bag from the saver, which, in turn, allows acting accordingly with the available media.

This study has limitations: a) in its development because, for being conducted in a reference center, it could underestimate the prevalence of the isolation of the *Staphylococcus epidermidis* strain in the saver, and b) in its context, because in terms of clinical meaning, many times it is difficult to establish which strains can be harmless commensals and which invasive pathogens.

## Conclusion

The microbiological characteristics of the red blood cells obtained after processing autologic blood recovered with the cell saver during heart surgery are of GRAM+ bacterial origin, the most isolated species being *Staphylococcus epidermidis*. Consequently, in order to reduce the presence of these GRAM+ cocci, an antibiotic should be added to the cell saver reservoir, according to a previously established protocol.
